# Risk of internal fixation treatment in intertrochanteric fracture based on different lateral femoral wall thickness: finite element analysis

**DOI:** 10.1186/s12891-024-07582-z

**Published:** 2024-06-13

**Authors:** Xu Zhang, Yazhong Zhang, Xiangyu Qi, Shaolong Huang, Yongxiang Lv, Wenbo Li, Chao Li, Ziqiang Zhu

**Affiliations:** 1 Department of Orthopaedics, The Second Affiliated Hospital of XuZhou Medical University, Xuzhou, 221000 China; 2 Graduate School of Xuzhou Medical University, Xuzhou, Jiangsu 221000 China

## Abstract

**Objective:**

The thickness of the lateral femoral wall, which is an important indicator for evaluating the stability and integrity of intertrochanteric fractures, has been widely studied in recent years. However, as a typical representative of internal fixation treatment, there are few reports on the biomechanical comparison between PFNA and DHS + CS. This study focused primarily on the biomechanical effects of different lateral femoral wall thicknesses on two types of internal fixation through finite element analysis.

**Methods:**

We randomly recruited a healthy adult and collected his femoral CT data to establish a model of femoral intertrochanteric fracture with different lateral femoral wall thicknesses. Following PFNA and DHS + CS fixation, femoral models were simulated, and variations in stress and displacement of the internal fixation and femoral head were recorded under the same physiological load.

**Results:**

First, finite element mechanical analysis revealed that the stress and displacement of the internal fixation and femoral head were lower in the femoral model after PFNA fixation than in the DHS + CS model. Second, as the outer wall thickness decreased, the stress and deformation endured by both types of internal fixation gradually increased.

**Conclusions:**

Finite element analysis determined that PFNA exhibits significantly better biomechanical stability than DHS + CS when subjected to varying lateral femoral wall thicknesses. Moreover, lateral femoral wall thickness substantially affects the stability of the two internal fixation biomechanical environments. When the thickness of the lateral femoral wall is too small, we do not recommend using extramedullary fixation because there is a significant risk of internal fixation fracture.

## Introduction

The incidence rate of hip fractures continues to rise with the aging of the population and the increase in osteoporosis patients, while the 30-year mortality rate for patients who have suffered a fracture remains at 5% [1]. Intertrochanteric femur fractures account for 40% of all hip fractures; their high incidence and mortality rates have made them an important public health problem [2, 3]. 

Currently, the clinical treatment of internal fixation in young patients includes:: anti-rotation proximal femoral nail (PFNA) and dynamic hip system with anti-rotation screw (DHS + CS) [4]. As a typical representative of intramedullary fixation, PFNA reduces surgical trauma, shortens surgical time, and enhances biomechanical stability. It also has a better anti-rotation effect and strong support ability [5]. As an external fixation system, DHS has numerous advantages, including low treatment cost and the ability to perform a dual function of compression and sliding, a strong structure that allows patients to walk with partial weight in the early stage. However, inherent biomechanical defects, including eccentric fixation, long arm length, high torque, and poor anti-rotation performance, result in poor treatment efficacy for unstable intertrochanteric fractures of the femur [6]. 

The concept of the lateral femoral wall was first proposed by an Israeli orthopedic physician, Yechiel Gotfried, in 2004 [7]. It is also recognized as one of the three major developments for treating intertrochanteric femur fractures in the past 20 years (namely, apical distance, cortical support, and lateral femoral wall). The lateral femoral wall thickness is defined as follows: on an X-ray anteroposterior film, a marker point is made 3 cm below the unnamed tubercle of the greater trochanter, and then a straight line is drawn at 135° along the femoral shaft, intersecting the fracture line between the trochanters at a specific point. The distance between these two points is the thickness of the lateral femoral wall [8]. The thickness of the lateral femoral wall reflects the integrity and stability of intertrochanteric fractures to some extent and is also related to the incidence of secondary fractures and complications [9]. Therefore, variations in the thickness of the lateral femoral wall invariably influence the biomechanical environment of internal fixation, potentially leading to different clinical outcomes and even failure of the procedure [3, 10]. 

Therefore, this study aimed to investigate the biomechanical effects of different lateral femoral wall thicknesses on PFNA and DHS + CS fixation of femoral intertrochanteric fractures using a finite element analysis model.

## Materials and methods

### Patient information and ethical review

We randomly recruited a volunteer who was a 55-year-old male, 175 cm tall and weighing 70 kg. X-ray examination ruled out hip joint diseases such as femoral head necrosis, dysplasia, and osteoporosis. After obtaining informed consent from the volunteer, a computed tomography scanner was used to scan their femur, and data were obtained. This study was approved by the hospital ethics committee.

### Modeling of the femur and internal fixation

**Fig. 1:**
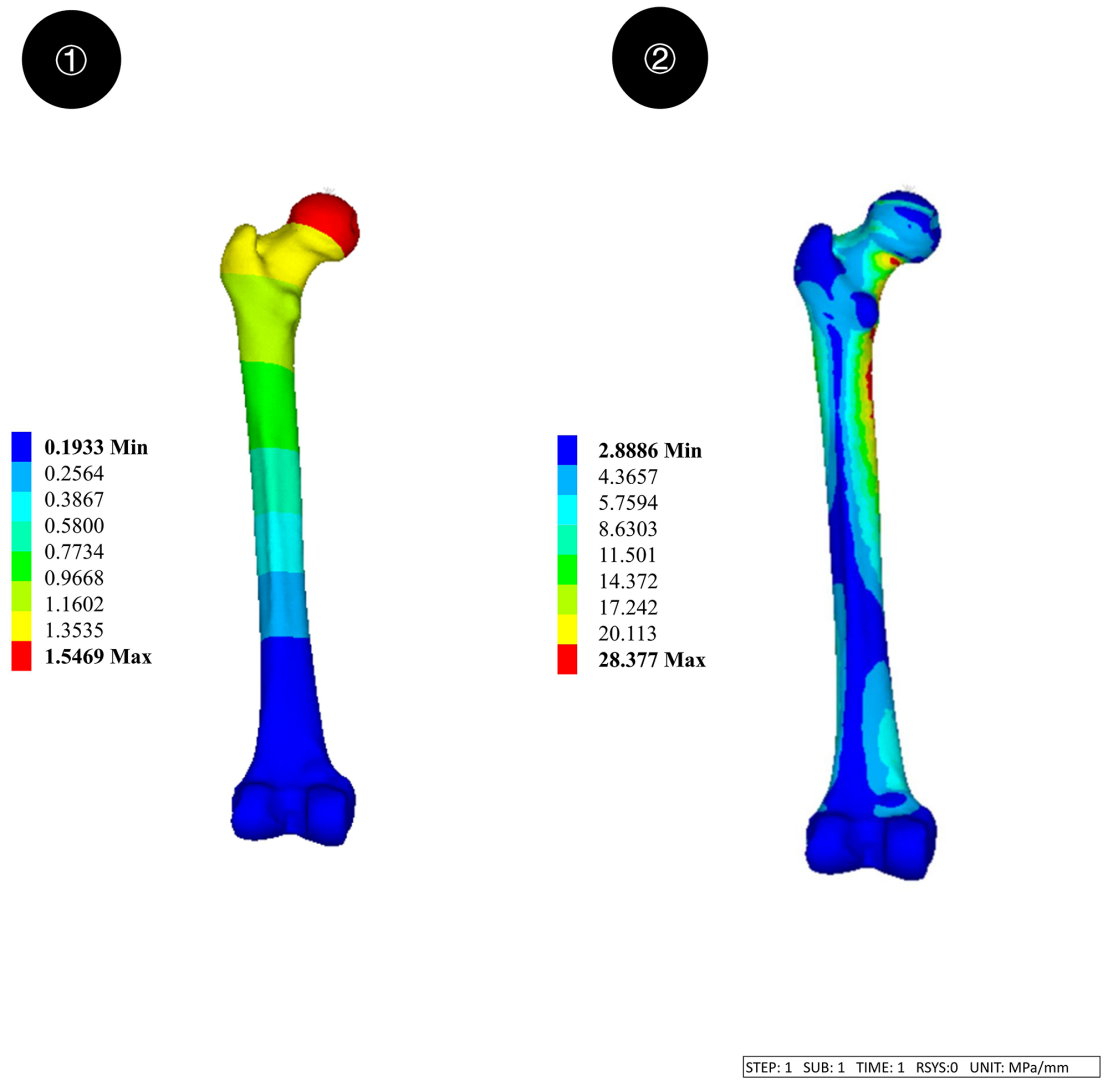
The results of the model validation. ①: The displacement of the femur ②: The stress of femur

**Fig. 2:**
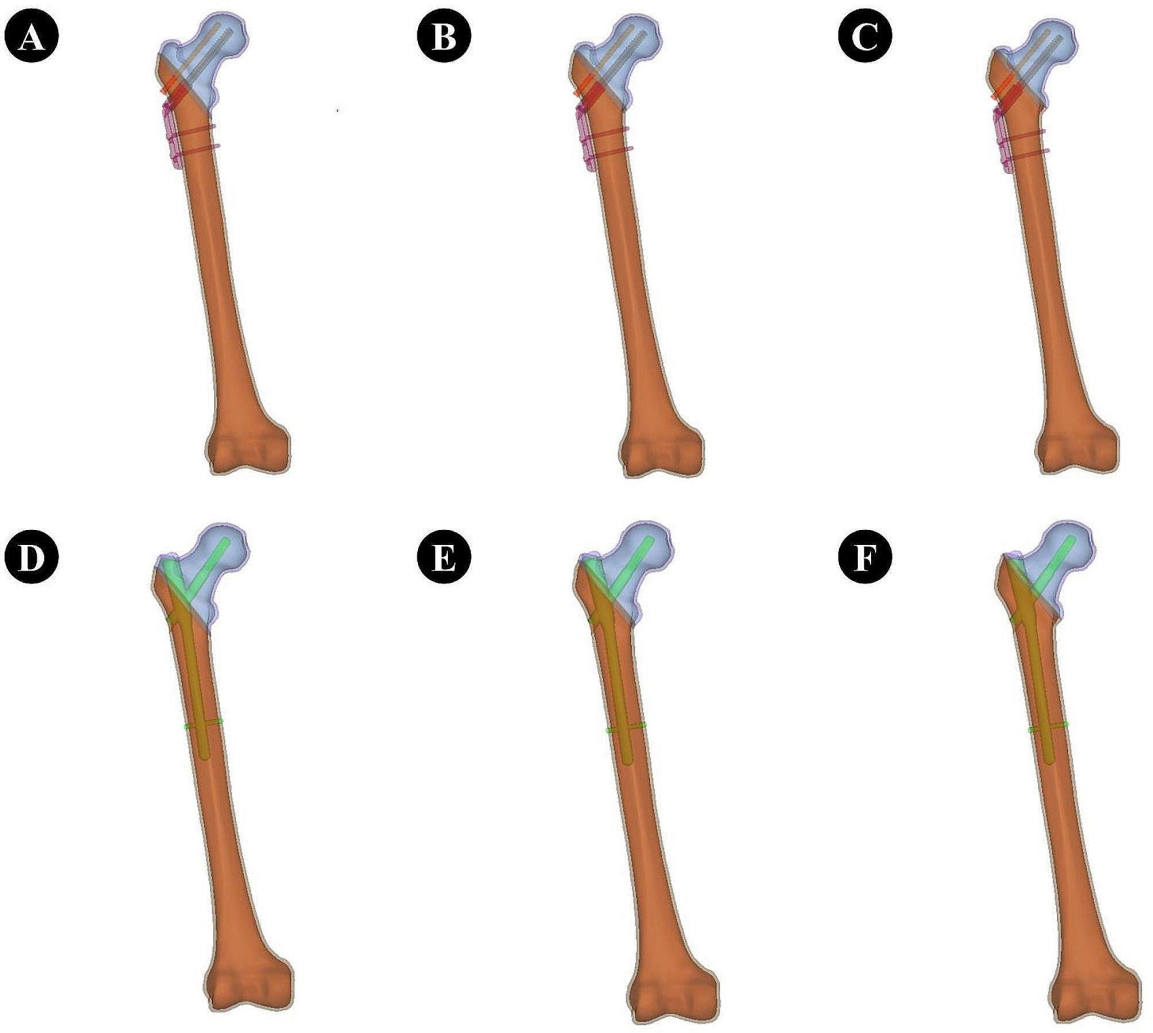
Finite element modeling diagram. **A-C** refers to the use of DHS + CS to fix femoral intertrochanteric fractures with different lateral wall thicknesses of 15.5 mm, 20.5 mm, and 25.5 mm, respectively. **D-F** refers to the use of PFNA to fix femoral intertrochanteric fractures with lateral wall thicknesses of 15.5 mm, 20.5 mm, and 25.5 mm, respectively

**Fig. 3:**
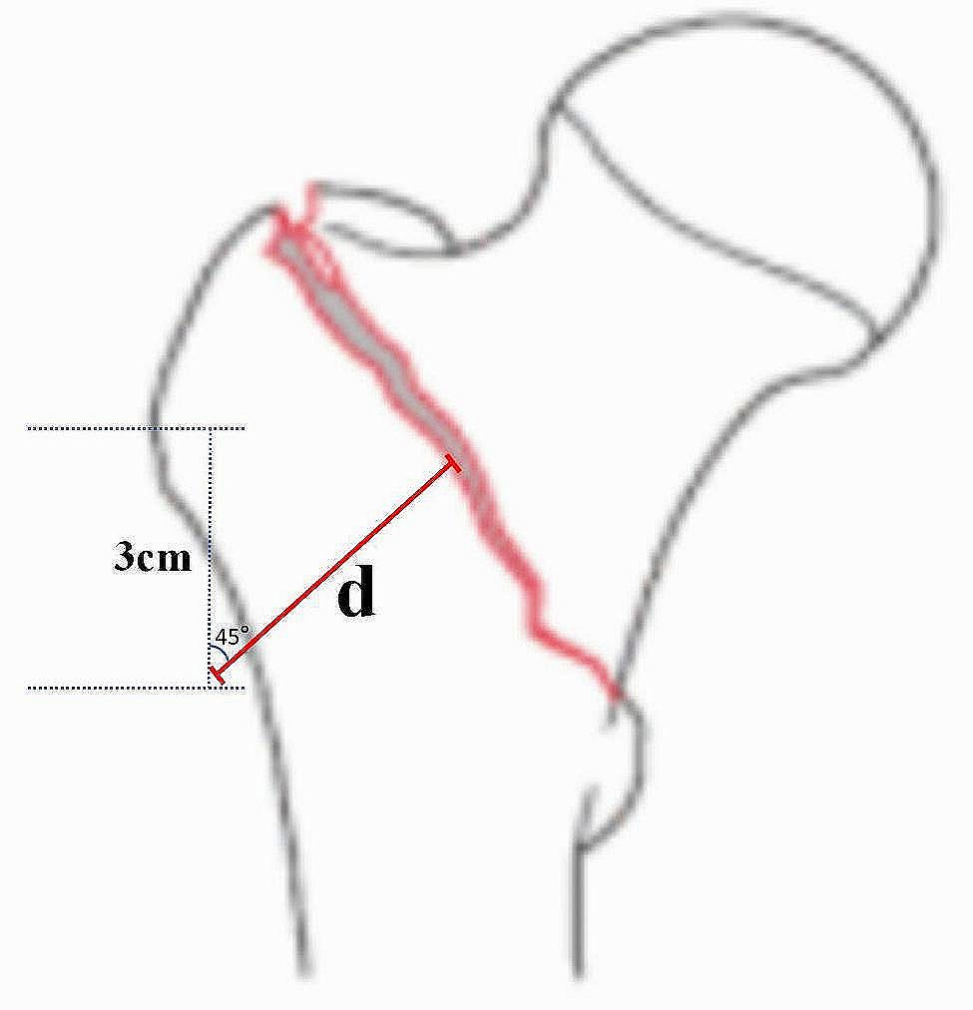
The location and size of the fracture in the bone. **d**: lateral femoral wall thicknesses

The 2D CT image data of the volunteer with a thickness of 0.625 mm was stored in the Dicom format and imported to the Mimics 21.0 medical image processing software for threshold segmentation, region growth, threshold editing, and 3D reconstruction for 3D modeling of the femur. The CT data we used are as follows: Width and Height:512 PX Pixel Size:0.970703 mm Field of View:497.00 mm Number of Slice:1195 Slice Thickness:0.900 mm. The generated file was saved in the STL format. Geomagic12.0 software was used to process the model mesh, extract curves, establish surfaces and grids, and fit surfaces to complete the establishment of the surface model. The above-mentioned surface model was imported into Creo Parametric 5.0.5.0 software to develop a 3D solid model. We validated the model to ensure its effectiveness. The results of our validation of the model are as follows: the compressive stiffness is 967.74 N/mm at a loading condition of 1500 N, which coincides with the results of Papini et al. with a compressive stiffness of (0.76 ± 0.26) KN/mm [11]. The results of the model validation are shown in Fig. 1. The PFNA and DHS + CS models were also developed using Creo Parametric 5.0.5.0 software. Finally, material properties were assigned to each part of the model using Hypermesh software. The PFNA we selected consists of a nail, a spiral blade, and a locking screw, whereas DHS + CS consists of a bone plate, metal bone screws, compression tail screws, and an anti-rotation screw. Three models of femoral intertrochanteric fractures with different LFW thicknesses (15.5, 20.5, and 25.5 mm, respectively) were established using SolidWorks. In the SolidWorks software, a plane is created, rotated and moved to a specific position, copied and pasted, and the pasted plane is kept parallel to the first plane using the parallel function in the assembly function of the software, and three different distances of 15.5, 20.5 and 25.5 mm are created, and Boolean reduction of the different planes to the same bone model is performed to model fractures of different lateral wall thicknesses. Two types of internal fixation were also Boolean reduced from the femoral model to complete the overall modelling of the post-fracture model. To ensure the sensitivity of the mesh, we carried out a mesh convergence study with a total of seven sets of analyses with different mesh sizes, the smallest being 1 mm and the largest being 5 mm, to study the deformation and stress distribution on the model under the seven mesh sizes. Through comparative analysis, considering that 1 mm mesh size is too small and the number of meshes is too high, which is too demanding on the capability of the analysis workstation, for all other mesh sizes, we found that when the mesh size is 2.5 mm, the size of the error is 1.6%, and therefore the best convergence is achieved by using this mesh size. Specific information on the mesh convergence study is shown in Table 1. Femoral models under different internal fixation are illustrated in Fig. 2. Figure 3 shows the location and size of the fracture in the bone. Element information consisting of finite elements models are shown in Table 2.

**Table 1:** Element information consisting of finite elements models

	Nodes	Elements
DHS + CS (lateral wall thicknesses15.5 mm)	1733906	1124640
DHS + CS (lateral wall thicknesses20.5 mm)	1727339	1119509
DHS + CS (lateral wall thicknesses25.5 mm)	1704169	1102199
PFNA (lateral wall thicknesses15.5 mm)	1550569	1023419
PFNA (lateral wall thicknesses20.5 mm)	1511921	994223
PFNA (lateral wall thicknesses25.5 mm)	1567650	1034280

**Table 2:** Parameters of elastic moduli and Poisson’s ratio

	Elastic modulus (MPa)	Poisson’s ratio
cortical bone	16800	0.30
cancellous bone	840	0.20
DHS + CS	110000	0.31
PFNA	110000	0.31

### Parameter settings

The titanium alloy materials were used in PFNA and DHS + CS, and the femur consists of cortical bone, cancellous bone, and a hollow medullary cavity. We assigned different elastic modulus and Poisson’s ratio parameters to them in Hypermesh software based on published research data: the cortical bone elastic modulus was 16,800 MPa, Poisson’s ratio was 0.30, the elastic modulus of cancellous bone was 840 MPa, and the Poisson’s ratio was 0.20, and the elastic modulus of PFNA and DHS + CS was 110,000 MPa with a Poisson’s ratio of 0.31 [12, 13]. Seen in Table 3.

**Table 3:** Information about mesh convergence

Mesh size(mm)	Displacement(mm)	VMS(MPa)	Error(%)	Nodes	Elements
1	2.17	41.31	0	9134151	6490950
1.5	2.17	40.57	1.8%	3491082	2457690
2	2.17	39.73	3.9%	1842459	1289577
2.5	2.16	40.64	1.6%	1374477	968385
3	2.16	39.58	4.3%	683985	470007
3.5	2.16	38.32	7.8%	443424	301395
5	2.16	38.13	6.6%	165323	106573

### Mechanical loads and boundary conditions

**Fig. 4:**
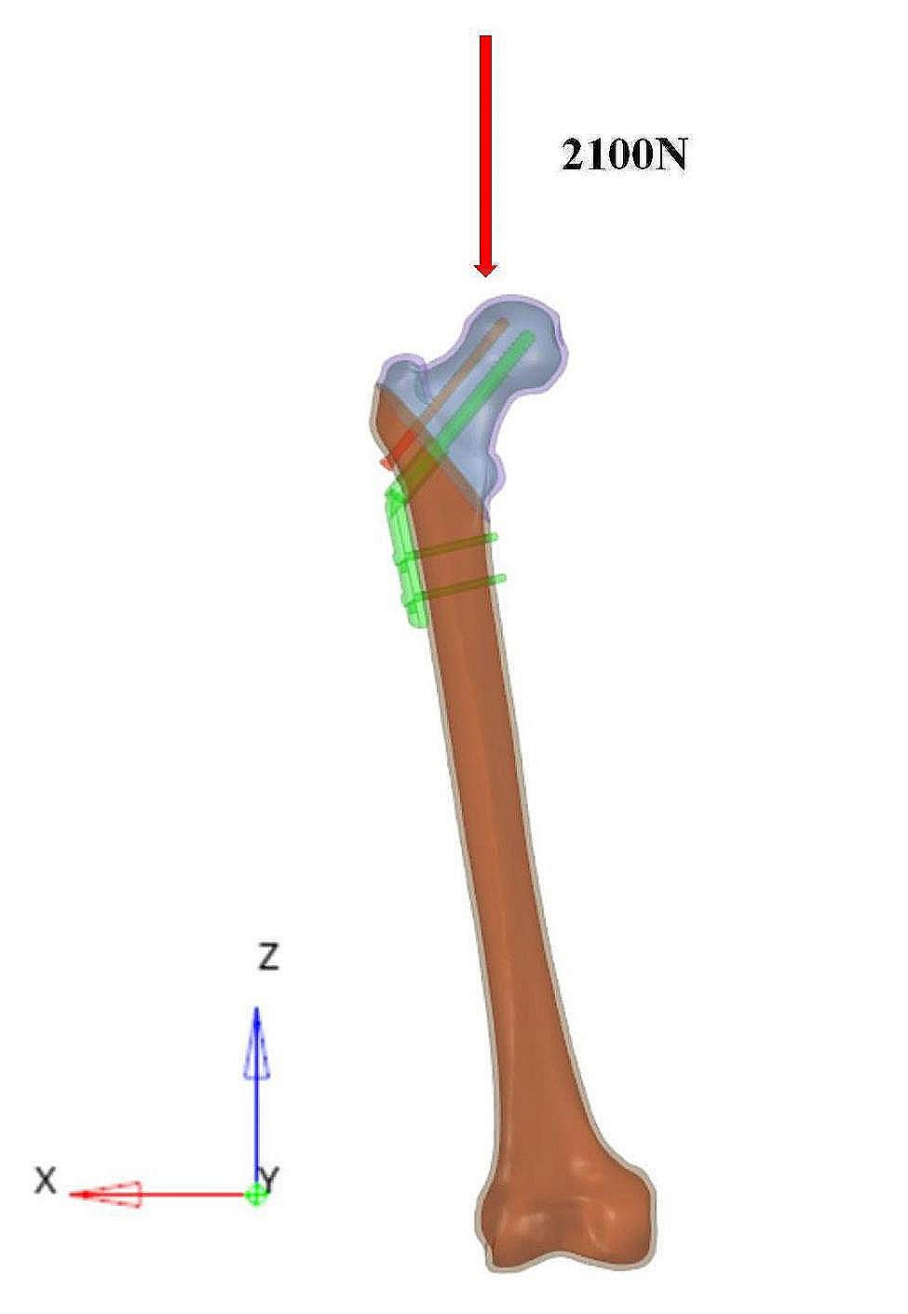
The loading direction of femur

All nodes at the distal end of the femur had degrees of freedom set to 0 in all six directions, and a load of three times the body weight (2100 N) was applied above the femoral head to simulate the load generated during normal gait cycles [14]. The loading direction is shown in Fig. 4. All interfaces between the implant and the two fracture ends were considered frictional contact, and the friction coefficients between the bone-bone, bone-implant, and implant-implant were set to 0.46, 0.30, and 0.23, respectively [15–17]. 

### Evaluation criterion

The maximum displacement value and peak von Mises stress were selected as indicators in the finite element analysis to evaluate the stability and risk of internal fixation failure of the two types of internal fixation devices for the femur. We also compared the von Mises stress and maximum displacement of the femoral head, femoral lateral femoral wall, and two types of internal fixation under the load generated during normal gait circulation in three different thickness models of the femoral lateral femoral wall (15.5, 20.5, and 25.5 mm).

## Results

### Stress

#### Dynamic hip system + anti-rotation screw (DHS + CS)

① When the thickness of the lateral femoral wall was 15.5, 20.5, and 25.5 mm, the stress of the femoral head was 204.65, 182.36, and 168.79 MPa, respectively.
② When the thickness of the lateral femoral wall was 15.5, 20.5, and 25.5 mm, the stress of internal fixation was 1065.65, 1096.94, and 941.74 MPa, respectively.

#### Anti-rotation proximal femoral intramedullary nail (PFNA)

① When the thickness of the lateral femoral wall was 15.5, 20.5, and 25.5 mm, the stress of the femoral head was 114, 94.18, and 82.74 MPa, respectively.
② When the thickness of the lateral femoral wall was 15.5, 20.5, and 25.5 mm, the stress of internal fixation was 446.73, 580.52, and 511.33 MPa, respectively.

**Fig. 5:**
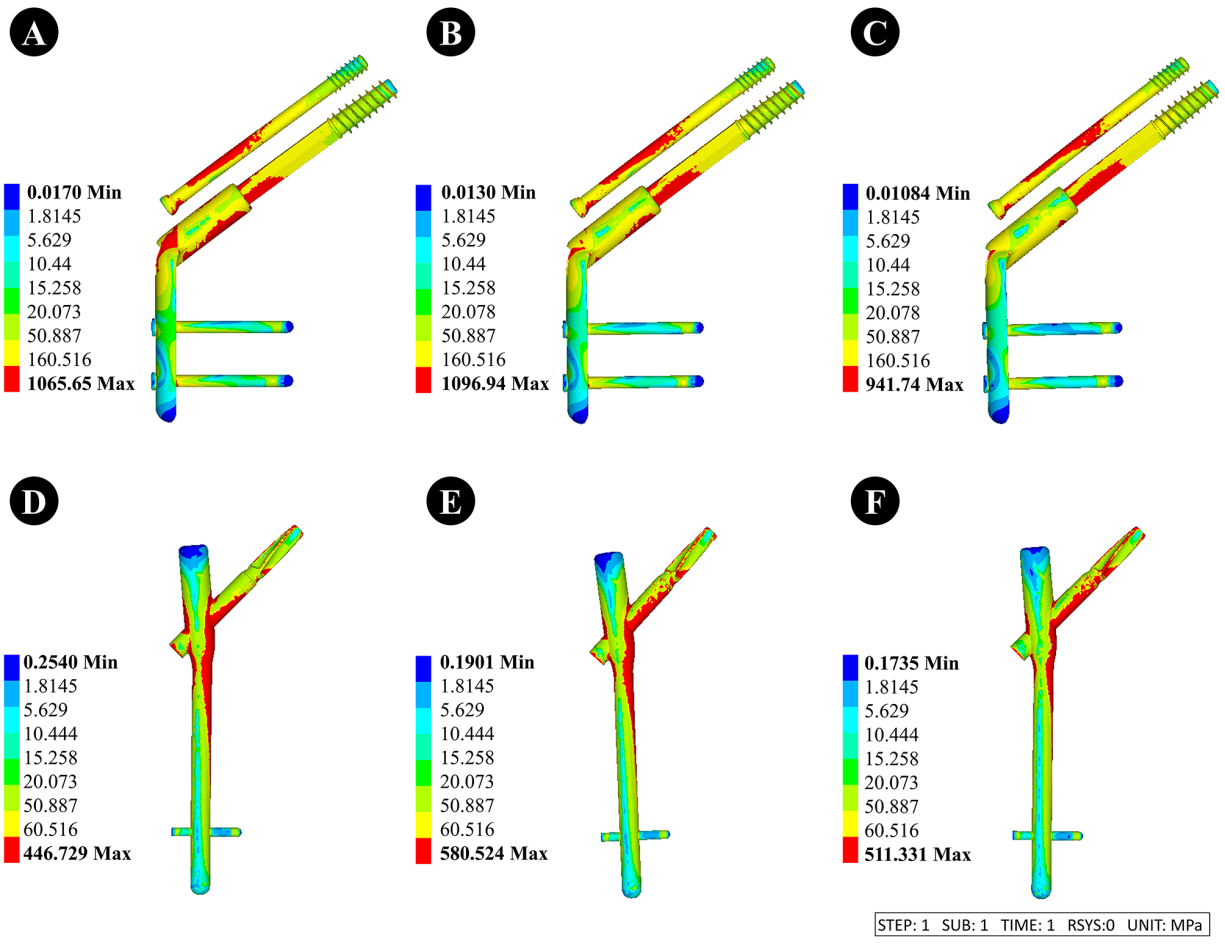
Stress nephogram of internal fixation with different lateral femoral wall thicknesses of 15.5 mm, 20.5 mm, and 25.5 mm for different types of internal fixation. **A-C** is fixed using DHS + CS, and **D-F** is fixed using PFNA

**Fig. 6:**
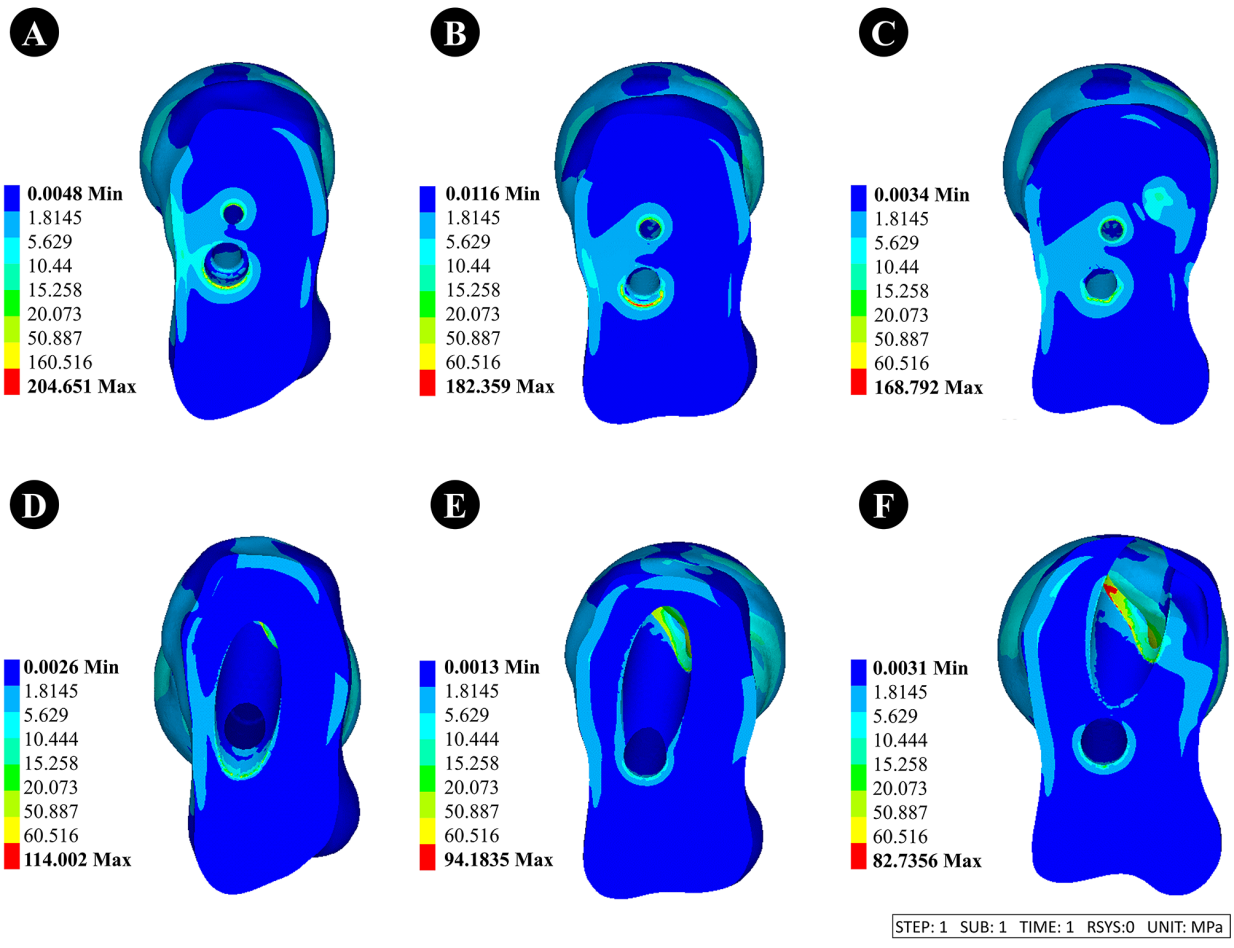
Stress nephogram of femoral head with different lateral femoral wall thicknesses of 15.5 mm, 20.5 mm, and 25.5 mm for internal fixation. **A-C** is fixed using DHS + CS, and **D-F** is fixed using PFNA

The stress nephogram of internal fixation and femoral head are displayed in Figs. 5 and 6.

### Displacement

#### Dynamic hip system + anti-rotation screw (DHS + CS)

① When the thickness of the lateral femoral wall was 15.5, 20.5, and 25.5 mm, the displacement of the femoral head was 4.72, 4.42, and 4.23 mm, respectively.
② When the thickness of the lateral femoral wall was 15.5, 20.5, and 25.5 mm, the displacement of internal fixation was 4.22, 3.95, and 3.78 mm, respectively.

#### Anti-rotation proximal femoral intramedullary nail (PFNA)

① When the thickness of the lateral femoral wall was 15.5, 20.5, and 25.5 mm, the displacement of the femoral head was 3.61, 3.47, and 3.36 mm, respectively.
② When the thickness of the lateral femoral wall was 15.5, 20.5, and 25.5 mm, the displacement of internal fixation was 3.29, 3.12, and 3.01 mm, respectively.

**Fig. 7:**
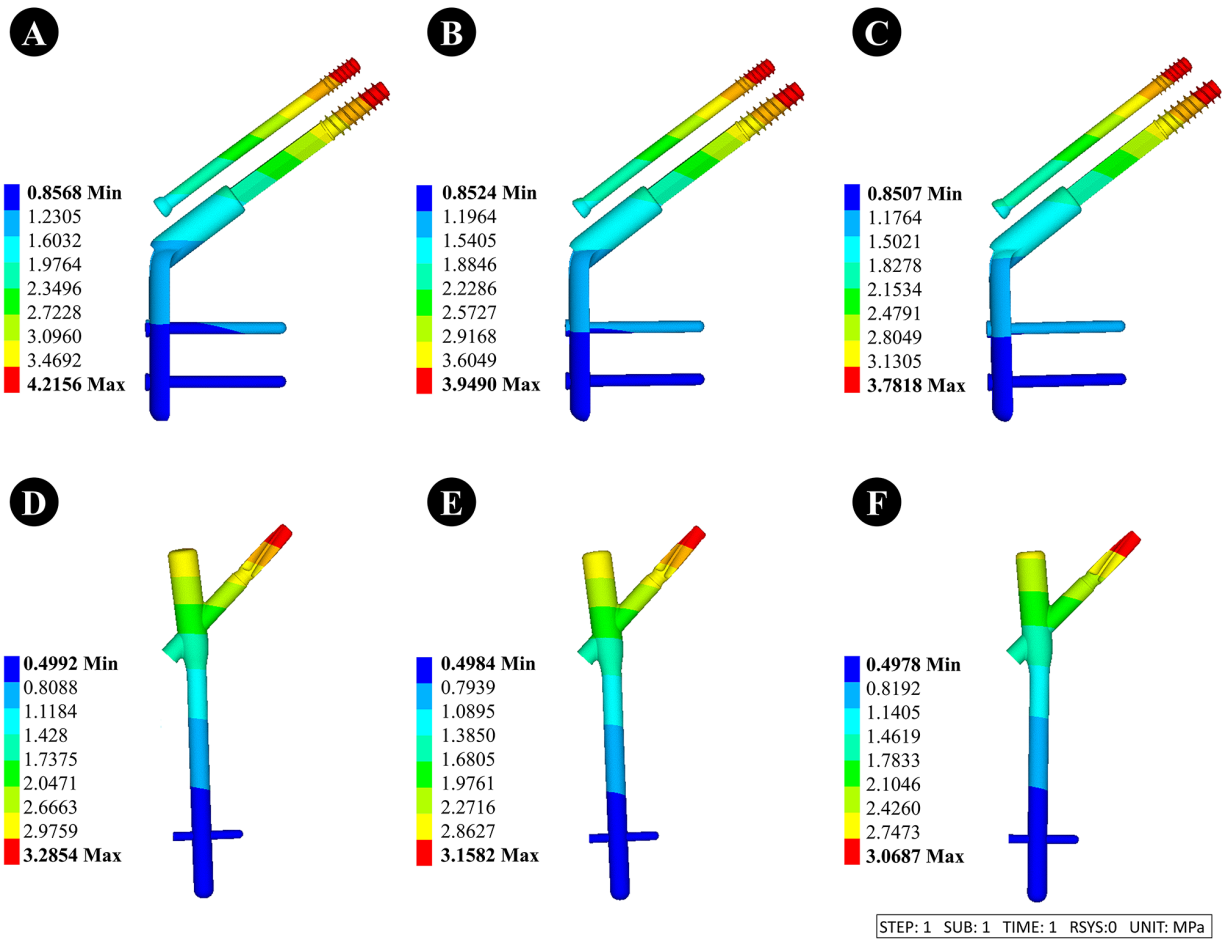
Internal fixation displacement maps with different lateral femoral wall thicknesses of 15.5 mm, 20.5 mm, and 25.5 mm, respectively. **A-C** is fixed using DHS + CS, and **D-F** is fixed using PFNA

**Fig. 8:**
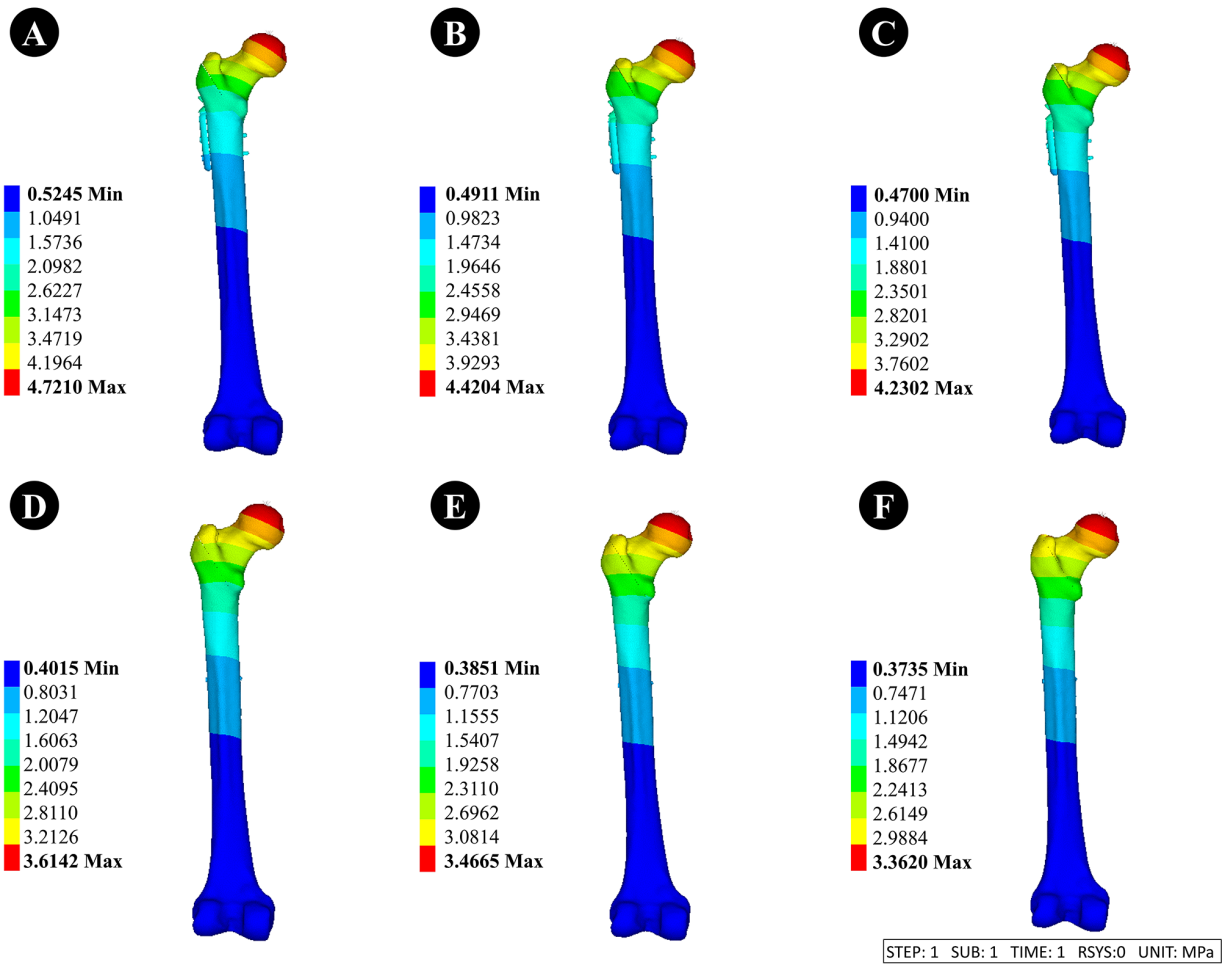
Femoral displacement maps with different lateral femoral wall thicknesses of 15.5 mm, 20.5 mm, and 25.5 mm for different internal fixation methods. **A-C** is fixed using DHS + CS, and **D-F** is fixed using PFNA

The displacement of internal fixation and femur are depicted in Figs. 7 and 8.

**Fig. 9:**
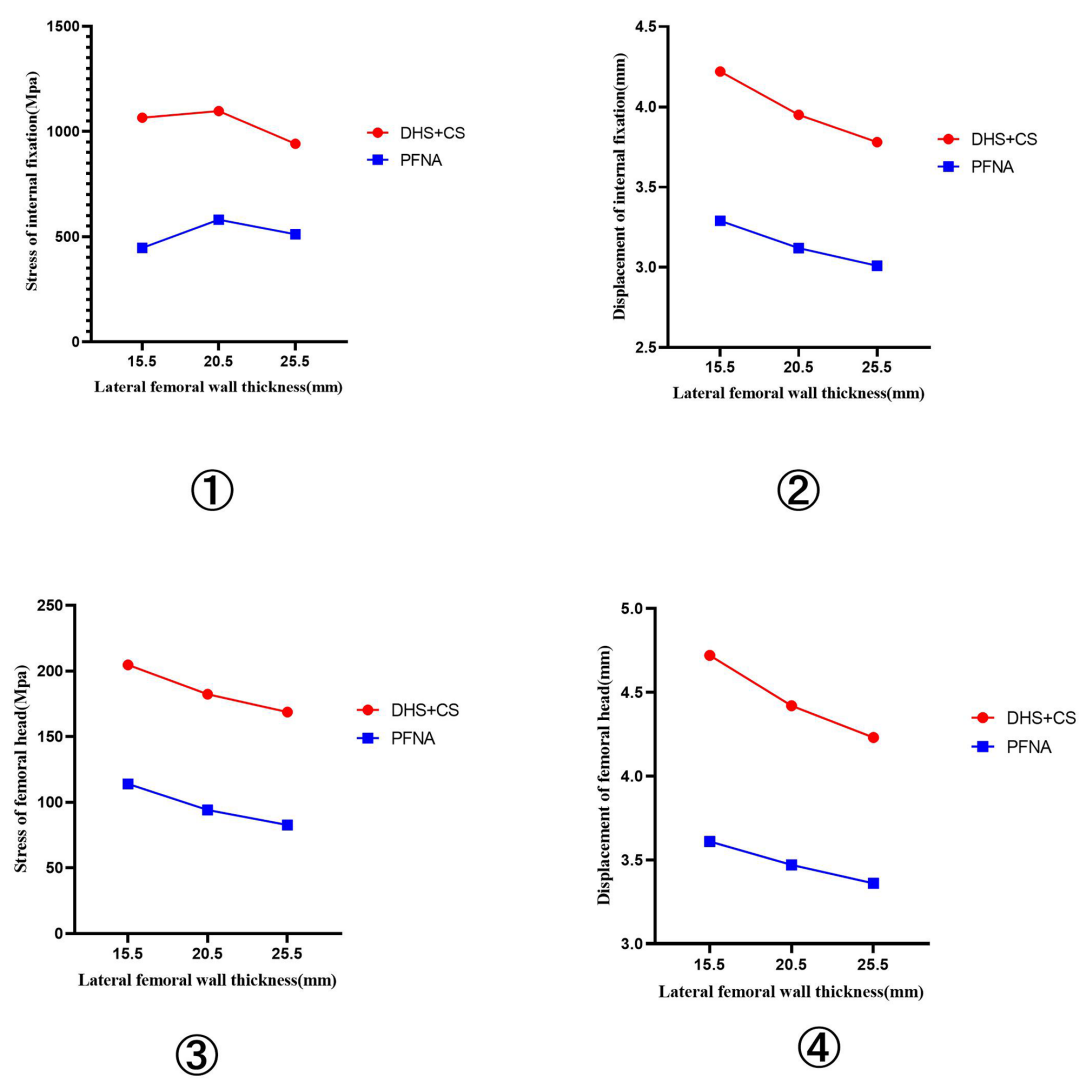
①:The maximum stress of internal fixation ②:The maximum displacement of internal fixation ③:The maximum stress of femoral head ④:The maximum displacement of femoral head

The maximum stress and displacement of internal fixation and femur are shown in Fig. 9.

## Discussion

Femoral intertrochanteric fracture, one of the fundamental issues in orthopedics, although often caused by low-energy injuries, can cause serious functional defects in patients. Its incidence rate exhibits an annual increase, whereas the mortality rate remains constant at 5% throughout the year [1, 18]. Proper and ideal internal fixation treatment for intertrochanteric femur fractures has always been a challenge for orthopedic doctors. Stable fixation of intertrochanteric femur fractures is the foundation for allowing patients to recover and bear weight early after surgery [19]. Presently, internal fixation surgery in clinical practice mainly includes two treatment options: intramedullary fixation and extramedullary fixation. The typical representative of intramedullary fixation is the widely used new type of internal fixation in recent years: anti-rotation proximal femoral nail (PFNA), whereas extramedullary fixation is the classic dynamic hip system (DHS) in clinical practice [20]. Although DHS has dual functions of compression and sliding and good shear resistance, its anti-rotation performance remains poor. In clinical use, the addition of an anti-rotation screw to improve stability is often necessary [21]. It is challenging to evaluate the impact of lateral femoral wall thickness on the biomechanical environment of the femur and internal fixation in clinical or in vivo studies. However, the gradual application of finite element analysis methods in the field of orthopedics has enabled the construction of three-dimensional finite element models of femoral intertrochanteric fractures that have been treated with various internal fixation methods. This facilitates a more convenient comparison of their sensitivity differences within the biomechanical environment [22]. 

Unstable intertrochanteric fractures of the femur usually involve the medial or lateral wall of the femur, thereby reducing the resistance of the femur to pressure. The medial wall of the femur is mainly composed of the lower end of the femoral neck, the lesser trochanter, and the femoral moment located at its depth. It is also a key factor in determining the stability, internal fixation efficacy, and prognosis of intertrochanteric fractures [3]. Nie et al. also showed that insufficient medial wall support can lead to poor femoral healing and hip varus deformity after surgery. Since its introduction by Dr. Gotfried in 2004, the concept of lateral femoral wall thickness has received increasing attention and research regarding the stability and prognosis of femoral intertrochanteric fractures [7, 23]. We simulated the biomechanical effects of different internal fixation treatments for intertrochanteric fractures of the femur by reconstructing finite element models with varying thicknesses of the lateral femoral wall.

When comparing DHS with PFNA in terms of clinical application, PFNA demonstrated several advantages, such as ease of operation, shorter surgical duration, early rehabilitation training for postoperative patients, and reduced bed rest time and complications [24, 25]. Notably, our study revealed that the peak von Mises stress of PFNA with intramedullary fixation decreased by 58.1%, 47.1%, and 45.7% compared with DHS + CS with extramedullary fixation, where the lateral femoral wall thickness measured 15.5, 20.5, and 25.5 mm, respectively. Although the von Mises stress for each model remains below the yield stress, increasing the maximum stress increases the risk of internal fixation fatigue failure. And the present model has been validated with the previous literatures with same material properties and boundary and loading conditions and the results of the present model were well collaborated with the previous one we use in other study [26]. Therefore, it is evident that PFNA has better stability and a lower risk of internal fixation fracture and failure than DHS. However in the context of Verification, Validation and Uncertainty Quantification (VVUQ), sensitivity analysis has become increasingly important. Therefore, the parameters utilized in this research are only appropriate for healthy adults, and alterations in these parameters for other fracture patients may yield varying outcomes.

Simultaneously, our study revealed that the thinner the lateral femoral wall, the greater the stress of DHS + CS and PFNA, indicating a higher risk of hip varus deformity, internal fixation fractures, and failure in intertrochanteric fractures of the femur after treatment with these two types of internal fixation methods [27]. Furthermore, our stress cloud map analysis also identified that the stress of PFNA is highly concentrated at the tail of the spiral blade and its junction with the intramedullary nail, which aligns with the findings reported by Zheng et al. [10]. The stress of DHS is concentrated near the interface between the main nail and the fracture line. This also indicates that using PFNA in conjunction with intramedullary nail devices allows for greater vertical load-bearing capacity on the femur compared with DHS and is less affected by the thinner lateral femoral wall, which is an unstable fracture factor. Simultaneously, it exhibits enhanced shear resistance, vertical load resistance, and rotational resistance. Therefore, intramedullary fixation with PFNA is recommended for patients with femoral intertrochanteric fractures characterized by thinner lateral femoral walls.

Therefore, in clinical practice, we believe that PFNA has better fixation strength and stability than DHS + CS in patients with intertrochanteric femoral fractures with a lateral wall thickness of 15.5–25.5 mm. The smaller the lateral wall thickness, the better the anti-shear and anti-rotation ability of PFNA fixed in the medullary cavity.

Our research also exhibited some limitations. The femur and internal fixation were set as homogeneous and continuous materials, which is different from the actual situation. The fracture end was also set to be smooth and continuous, which is different from the actual one. Second, we simplified the mechanical effects of muscle attachment and ligaments surrounding the femur [28, 29]. The primary objective of this study was to evaluate the biomechanical performance of PFNA and DHS + CS internal fixation methods for treating femoral intertrochanteric fractures with different LFW thicknesses by constructing a finite element model. Further practical biomechanical experiments are needed to validate the outcomes of our research. And studying the femur in all its anatomical forms is a huge and complex project, which is also our next research direction and goal.

## Data Availability

The datasets used and/or analysed during the current study available from the corresponding author on reasonable request.
